# A Review of Federated Large Language Models for Industry 4.0

**DOI:** 10.3390/s26041116

**Published:** 2026-02-09

**Authors:** Feng Jing, Yujing Zhang, Mei Gao, Xiongtao Zhang, Huaizhe Zhou

**Affiliations:** Test Center, National University of Defense Technology, Xi’an 710106, Chinayujing_zhang_xd@163.com (Y.Z.); huaizhezhou@nudt.edu.cn (H.Z.)

**Keywords:** industry 4.0, large language model, federated learning

## Abstract

Industry 4.0 envisions a highly interconnected, autonomous manufacturing ecosystem enabled by the Industrial Internet of Things, Cyber-Physical Systems, and Artificial Intelligence. The emergence of large language models introduces new capabilities for semantic-aware decision-making, cross-domain knowledge integration, and intelligent automation. However, privacy, security, and regulatory constraints often isolate industrial data, impeding the scalability of LLMs in manufacturing. Federated learning addresses this by enabling decentralized LLM optimization without exposing raw data. This paper presents a comprehensive review of recent federated large language model research with a focus on industrial feasibility, comparing enabling techniques, system designs, and deployment strategies. Based on existing studies, forward-looking analyses are provided to highlight potential challenges and trade-offs in practical adoption, including computation and communication overheads, synchronization in large-scale federations, and system robustness. By bridging foundational methods with emerging industrial scenarios, we finally discuss the significant challenges associated with deploying federated large language models in complex industrial environments and outline a future research agenda.

## 1. Introduction

The core objective of Industry 4.0 is to establish a highly connected, data-driven intelligent ecosystem. This ecosystem integrates a suite of state-of-the-art technologies, including the Industrial Internet of Things (IIoT), Cyber-Physical Systems (CPS), and Artificial Intelligence (AI), which together provide the foundational support for advanced industrial operations. Its primary goal is to interconnect various industrial processes, such as product design, manufacturing, operations, and maintenance, thereby transforming production systems from automation-based frameworks into intelligent systems.

**Figure 1:**
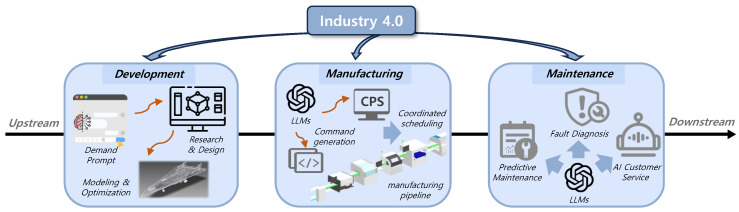
The application of LLMs in Industry 4.0. LLMs have progressively permeated various stages of industrial production, playing a driving role in product design optimization, SCM, and PdM.

To facilitate this transformation, AI must deliver an expanded and more comprehensive set of capabilities. In recent years, large language models (LLMs) have emerged as a significant breakthrough in AI. Supported by high-quality data and enhanced computational resources, these models are continually scaling in terms of parameters, which has greatly improved their context understanding and text generation abilities. Building a series of industrial agents based on LLMs has thus become a primary strategy for achieving the objectives of Industry 4.0. As illustrated in Figure 1, LLMs are increasingly integrated across various stages of industrial production, playing a pivotal role in product design optimization, supply chain management (SCM), and predictive maintenance (PdM) [1].

The high-quality data generated in these processes constitutes a critical resource for the continuous improvement of LLMs. As vertical specialization in LLMs becomes increasingly prevalent, there is a growing demand for training data that is not only professional and timely but also diverse. Moreover, with the proliferation of the IIoT and the enhancement of equipment intelligence, industrial data now exhibits highly dynamic and real-time streaming characteristics [2]. Traditional LLMs frequently rely on centralized learning strategies to extract knowledge from such data, requiring the consolidation of large datasets on cloud or centralized servers for unified training to achieve globally optimal models [3].

While centralized learning can yield high model performance under conditions of sufficient data scale and stable network environments, in the context of Industry 4.0, data is often dispersed across various factories, organizations, and companies. This dispersion frequently includes sensitive information, such as production processes and supply chain strategies, thereby posing significant risks concerning data security and privacy. Furthermore, conventional centralized learning struggles to meet the requirements for real-time learning; not only does the transfer of large amounts of data heighten privacy and compliance risks, it also entails substantial communication overhead and delays in updating models. As a result, the typical barriers to data sharing among companies frequently lead to pronounced data silos, and the lack of effective sharing mechanisms in the industrial data environment has emerged as a key constraint in the development of industrial large models.

Federated learning (FL) is an emerging decentralized training paradigm that provides a comprehensive framework to address significant challenges. Its architecture restricts the exchange to model parameters between central servers and clients, thereby ensuring that sensitive data remains securely stored on local devices. Each client performs iterative local optimization using newly generated data, while aggregation mechanisms alleviate distribution discrepancies among nodes to facilitate robust global model updates. Consequently, FL fosters the continual integration of knowledge across diverse domains and enhances overall model generalization.

Integrating LLMs with FL to establish a digital federation for Industry 4.0 represents a promising and logical progression. As summarized in Table 1, most existing literature reviews have predominantly focused on the isolated themes of “LLM + Industry”, “FL + Industry”, or “LLM + FL”.

Research on the integrated application of LLMs and FL in industrial environments remains in its early stages, particularly regarding systematic explorations of their combined potential to address data silos and heterogeneity within the Industry 4.0 paradigm. Specifically, Chkirbene et al. [3] concentrated on the use of general-purpose LLMs in particular industries, evaluating the technological development of LLM and exploring use cases across industries in decision-making, automation, and content production, including healthcare, finance, and customer support. Raza et al. [4] focused on the applications and challenges of LLM in a variety of sectors, particularly with regard to security, privacy, and ethical issues. Other related studies also relate to LLM applications in digital manufacturing and smart factories [9,10] but offer only negligible coverage on FL programs to address data silos and protect privacy. Cheng et al. [5] explored motivations and challenges in combining FL with LLM but did not cover industrial scenarios. Syed et al. [6] and Leng et al. [7] investigated and assessed the use of FL as a response to challenges of data privacy and security in manufacturing settings of Industry 4.0, but without an organized conversation on its relation to decisions involving LLM. Yang et al. [8] discussed combining IoT, LLM, and FL to solve privacy protection issues, but their work was confined to specific application scenarios. Overall, these studies do not provide a comprehensive framework summarizing the integrated application of LLM and FL in Industry 4.0.

It is also critical to acknowledge that employing LLMs in conjunction with FL can introduce adverse effects and novel challenges. For example, the extensive parameter scales associated with LLMs may lead to communication bottlenecks and synchronization delays in FL environments [11]. The typical non-independent and identically distributed (non-IID) characteristics of industrial data can also cause knowledge drift during model updates [12]. Information leakage and privacy inference during the transmission phases of model parameters or gradients are also critical concerns that require urgent consideration [13]. Neglecting these issues could undermine the trustworthiness and security of industrial intelligent systems.

In this paper, we analyze the complementary mechanisms, representative use cases, and key challenges associated with integrating LLM with FL in the context of Industry 4.0. Building upon existing studies, we further provide prospective insights into the feasibility of deploying a federated large language model (Fed-LLM) in real-world industrial environments. The main contributions of this paper are summarized as follows:

- We provide a structured overview of the research landscape at the intersection of LLMs and FL for industrial applications, clarifying the motivation for their integration and analyzing their potential to address key challenges in large-model deployment under Industry 4.0 constraints.
- We review and comparatively analyze representative Fed-LLM techniques and system architectures from both algorithmic and system perspectives, highlighting how different design choices impact industrial feasibility.
- We summarize upstream-to-downstream industrial application scenarios enabled by the integration of LLMs and FL and discuss representative use cases to illustrate practical workflows and deployment patterns.
- We identify critical challenges that hinder large-scale industrial adoption of Fed-LLM and outline promising directions for future research.

**Figure 2:**
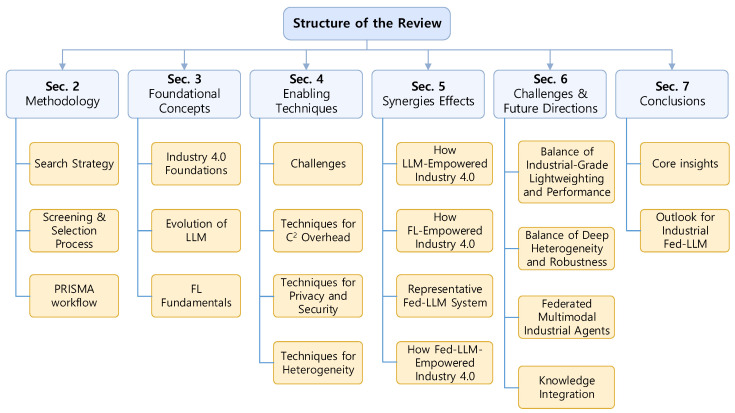
Structure of this review. The diagram outlines the paper’s progression through its main sections: Methodology (Section 2), Foundational Concepts (Section 3), Enabling Techniques (Section 4), Synergistic Effects (Section 5), Challenges and Future Directions (Section 6), Conclusions (Section 7). Key topics within each section are indicated, offering a roadmap for the reader.

As summarized in Figure 2, this paper is organized into the following sections. Section 2 describes the literature retrieval and selection process methodology adopted in this study. Section 3 introduces the foundational concepts of Industry 4.0, FL and LLM. Section 4 examines representative Fed-LLM techniques, focusing on communication efficiency, privacy and security, and heterogeneity challenges. Section 5 reviews the synergistic integration of LLMs and FL in Industry 4.0, including enabling technologies, systems, and application scenarios. Section 6 discusses the key challenges and future research directions for deploying Fed-LLMs in real-world industrial environments. Finally, Section 7 concludes the paper by summarizing the main insights and perspectives.

## 2. Methodology

In order to ensure the standardization and comprehensiveness of the process of literature retrieval, selection and synthesis, this review follows PRISMA (Preferred Reporting Items for Systematic Reviews and Meta-Analyses) Guidelines. The research objectives, core research issues, inclusion and exclusion criteria and literature screening process were clarified.

### 2.1. Identification

This review focuses on the researches work of combining FL with LLM in the context of industry 4.0. To ensure both completeness and methodological rigor, the literature search in this review was intentionally designed using multiple keyword combinations rather than restricting the query to the strict intersection of FL, LLM, and Industry 4.0. Although studies explicitly addressing the joint integration of FL and LLMs in industrial scenarios represent the core focus of this review, preliminary investigations revealed that such three-way combined studies remain relatively limited due to the emerging nature of the topic.

Consequently, four complementary keyword combinations were adopted:

- FL and LLM: capturing foundational algorithmic frameworks and system-level designs for Fed-LLM;
- FL in Industry: covering mature federated learning solutions addressing industrial data privacy, communication efficiency, and edge deployment constraints;
- LLM in Industry: identifying industrial-oriented adaptations and applications of LLM;
- Fed-LLM in Industry: targeting studies that explicitly integrate all three dimensions.

This multi-stage search strategy enables the review to bridge foundational techniques and emerging integrated solutions. While a larger number of publications were retrieved from single-technology or dual-combination searches, only studies that provided direct methodological relevance, industrial applicability, or empirical insights supporting Fed-LLM integration were retained in the final synthesis.

We focus on the relevant literature involving the algorithm framework, practical industrial applications, key challenges and future research directions. We selected keywords as follows:

- Terms related to FL: federated learning, FL;
- Terms related to large-scale model: large language model, LLM;
- Industrial and information physical system scenarios: Industry 4.0, manufacturing;
- Terms related to mentioned challenges: resource constrained, communication, aggregation security, heterogeneity.

Boolean operators were used to combine the above terms within the title, abstract, and keyword fields. Based on the predefined keyword combinations, a structured literature search was conducted across multiple mainstream academic databases, including Web of Science, IEEE Xplore Digital Library, ACM Digital Library, Scopus, and arXiv (for preprints), covering publications from January 2023 to October 2025.

To enhance transparency and reproducibility, the specific search strings applied to each database are explicitly reported in Table 2. These database-specific queries were formulated in accordance with the indexing rules and query syntax of each platform, while maintaining consistent semantic coverage across different sources.

Peer-reviewed journal and conference publications were prioritized throughout the literature selection process. Preprints from arXiv were considered only when they were highly relevant to the core topic of Fed-LLM in industrial scenarios and when no suitable peer-reviewed alternatives were available at the time of retrieval. This inclusion is justified by the rapidly evolving nature of FL and LLM, where novel frameworks, system architectures, and industrial applications are often first disseminated as preprints prior to formal publication. To address potential quality concerns, all included preprints underwent the same full-text assessment and methodological relevance evaluation as peer-reviewed studies.

### 2.2. Exclusion and Inclusion Criteria

To ensure a focused selection of studies relevant to Fed-LLM in Industry 4.0, explicit inclusion and exclusion criteria were applied to progressively refine the initially retrieved literature. As summarized in Table 3, particular emphasis was placed on the feasibility of deploying FL- or LLM-based methods under realistic industrial constraints. Studies presenting purely conceptual, illustrative, or laboratory-level analyses were therefore excluded.

Specifically, publications were removed if FL or LLM were discussed only at an abstract or algorithmic level. In such cases, industrial deployment considerations—such as limited computation and communication resources, heterogeneity, privacy and security requirements—were not adequately addressed. In addition, to avoid scope dilution, studies in which FL or LLMs appeared merely as background concepts, comparative baselines, or auxiliary tools were excluded. Only works providing sufficient technical depth were retained, including those offering architectural descriptions, methodological details, or empirical validation.

### 2.3. Data Collection Process

**Figure 3:**
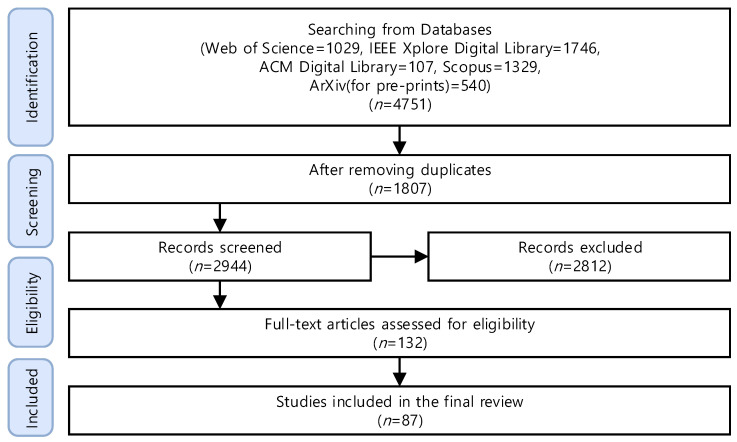
PRISMA flowchart.

In this study, we adopted a structured literature review process to organize the identification, screening, and analysis of relevant literatures. The complete data collection and screening process is summarized in the PRISMA flow diagram in Figure 3.

The initial literature search retrieved 4751 records: 1029 from Web of Science, 1746 from IEEE Xplore, 107 from ACM Digital Library, 1329 from Scopus, and 540 from ArXiv. After removing 1807 duplicates, 2944 unique records remained for screening. Titles and abstracts were carefully examined for relevance to FL, LLM, and their applications in Industry 4.0, resulting in the exclusion of 2812 records.

The remaining 132 articles underwent full-text assessment against predefined inclusion and exclusion criteria, focusing on technical depth and relevance to realistic industrial constraints. Following this evaluation, 87 studies were deemed eligible and included in the final review. Screening was conducted independently by two authors, with discrepancies resolved through discussion to minimize bias.

The included studies provide substantial insights into privacy protection, deployment on resource-constrained edge devices, and future research directions in Fed-LLM for Industry 4.0.

## 3. Foundational Concepts

In this section, we present the underlying concepts of Industry 4.0 and related technologies, a background overview of LLM, and the foundations of FL technologies.

### 3.1. Industry 4.0

Industry 4.0 signifies a profound transformation in manufacturing from automation toward intelligent and interconnected systems. Its core objective is to establish a self-aware, self-decision-making, and adaptive smart manufacturing ecosystem composed of the IIoT, CPS, and AI [14]. By establishing information connectivity between devices, factories, and supply chains, Industry 4.0 aims to transition from centralized production control to distributed intelligent decision-making.

#### 3.1.1. Industrial Internet of Things

The Internet of Things (IoT) achieves seamless connectivity between the physical world and the digital realm through the synergy of sensors, communication modules, and cloud platforms [15]. IoT-enabled industrial machinery is capable of collecting crucial operational data such as status, energy usage, temperature, and vibration. These data can then be transmitted in real time to central or edge computing nodes for analysis.

Using this premise, the IIoT has emerged as an important technology for smart manufacturing. It allows production facilities to build a data ecosystem that seamlessly connects the perception layer and the application layer, ultimately forming a smart factory system that is capable of sensing, communicating, and self-learning. The IIoT enables the real-time data collection of the entire production process, generating huge industrial datasets that can serve as an important basis for model training [16].

Nonetheless, heterogeneous device types, communication that varies over time and privacy restrictions of IIoT make traditional centralized training difficult to use in practice. Therefore, FL is seen as a key enabler of IIoT. It allows devices to train a model in a decentralized manner on their own local data by sharing only the updates to model parameters instead of sharing the data itself, allowing distributed learning without leaving the domain of the data itself [14]. IIoT and FL together permit multi-node collaborative learning, which is done without raw data aggregation and therefore, it can eliminate data silos. The distribution of computational and communication resources allows for heterogeneous network structures to be like IIoT for improved flexibility, robustness, and scalability in manufacturing systems.

#### 3.1.2. Cyber-Physical Systems

CPSs are a key aspect of the Industry 4.0 paradigm by tightly coupling computing, communication, and physical processes into a system architecture. CPSs consist of layers of perception, networking, computation, control, and actuation, which facilitate sensing, transferring data to computational layers on a network, and applying the computer’s decision-making back to the physical world for intelligent control.

The five-stage CPS architecture (5C Architecture) has five tiers: Connection, Conversion, Cyber, Cognition, and Configuration [17], proposing a systematic method for structuring intelligent manufacturing systems. In this architecture, the Connection and Conversion tiers, being much deeper, acquire and preprocess data from equipment, sensors, and control systems. The Cyber tier then enables data sharing, and collaboration of models, on the cloud, edge, and device tiers. In turn, the Cognition layer employs AI and large-scale models to deduce actionable insight and perform intelligent decision-making using the data. Finally, the Configuration layer will make system self-optimization and reconfiguration possible, through knowledge-based feedback.

In industrial contexts, CPSs installed on production lines allow for real-time tracking of the status of equipment and for predicting system irregularities. They also facilitate automatic changes to operating settings without human involvement. These features enable PdM and adaptive production capabilities. The presence of CPSs enhances the efficiency and safety of systems. They also give industrial processes the ability to learn continuously and dynamically optimize systems. CPSs provide the technological base for autonomous factories and reliable supply chains.

### 3.2. Large Language Model

LLMs represent a significant advancement in natural language processing (NLP), increasing generalization and reasoning by scaling model parameters and pre-training data. Studies have shown that, as parameter counts increase, models exhibit a consistent improvement trend on a number of downstream tasks. When the model is scaled sufficiently, “emergent abilities” occur, i.e., the model shows strong adaptive reasoning and few-shot learning abilities [18] on tasks for which it was never trained. Prominent examples include OpenAI’s GPT-3 (175 billion parameters) and Google’s PaLM (540 billion parameters), which have demonstrated an unprecedented depth of capabilities that far exceeds traditional pre-trained language models, such as BERT and GPT-2, in semantic understanding, logical reasoning, and on cross-task transfer.

From the standpoint of technological evolution, the field of language models has evolved through four stages: statistical modeling, neural modeling, pre-training, and large-scale autoregressive modeling. Earlier models were limited-domain, probabilistic statistics that models measured that. In contrast, LLMs using the Transformer architecture, achieved hierarchical modeling of both linguistic structure and semantic information through deep stacking and self-attention mechanisms. This architecture allowed models to handle long-range dependencies and generate semantically coherent text. The landscape of LLM has continued to evolve, with the current mainstream LLM technology represented by a number of examples, such as the GPT series used for text generation and dialogue, BERT and RoBERTa specializing in contextual understanding and classification tasks, PaLM and T5 supporting multi-task transfer learning, and the LLaMA series explicitly optimized for research or lightweight deployment.

The outstanding capability of LLM has prompted intelligent transformations across areas such as conversational AI, automated programming, legal analysis, diagnosis, and content development. Nevertheless, major computational burden and lack of transparency within training data remain significant challenges in ongoing research. Thus, efficiency for adapting LLM to specific industrial contexts—while maintaining interpretability and assurance of privacy—has become an important direction for research moving forward.

### 3.3. Federated Learning

As IoT and CPS become more widely used under Industry 4.0, issues of data privacy and system heterogeneity have become more pronounced. FL, which is a distributed collaborative modeling mechanism [19], has quickly become a prominent technological pathway for bridging smart manufacturing and industrial AI.

FL is a distributed machine learning methodology that safeguards privacy and personal data and generalizes LLMs to a host of downstream tasks while respecting privacy requirements. Unlike traditional approaches that rely on centralization, where sensitive data are uploaded to a central server where aggregation occurs, FL allows clients to train models locally on their private datasets with only the model updates uploaded to the central server. This mitigates the risk of data leaking with the aggregated data.

In a typical federated optimization process, the server performs weighted averaging on locally trained model parameters uploaded by clients using the FedAvg algorithm [20]:

(1) $$\theta_{t+1}=\sum_{i=1}^{N}\frac{n_{i}}{n}\theta_{t}^{i}$$

$\theta_{t}^{i}$ represents the model parameters of client *i* after the *t*th training round, $n_{i}$ denotes the client’s data volume, and $\theta_{t+1}$ indicates the global model parameters after the t+1th update. FedAvg achieves global optimization through simple weighted aggregation, ensuring both client data privacy and overall model performance improvement. In industrial applications of LLM, FedAvg and its variants offer essential support for model fine-tuning and personalized deployment. They enable these capabilities across distributed edge nodes and enterprise private data environments.

## 4. Enabling Techniques of FL and LLM in Industry 4.0

With the deep integration of LLM and FL, the application of Fed-LLM in industrial scenarios is gradually moving from theory to practice. In this section, we introduce the challenges and enabling techniques for deploying Fed-LLM in industrial contexts.

### 4.1. Challenges

**Figure 4:**
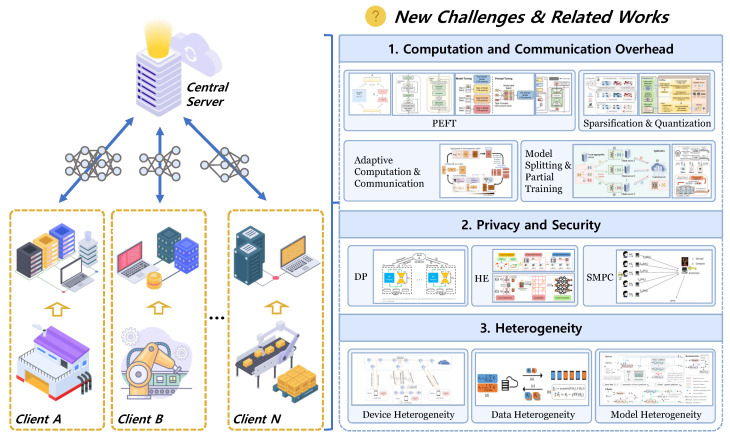
Fed-LLM challenges and related works in Industry 4.0. The application of Fed-LLM faces numerous challenges in industrial environments characterized by constrained C^2^ resources, data privacy and security, and strongly heterogeneous.

Although FL enables large models to collaboratively achieve model improvement while ensuring privacy protection [21], the application of Fed-LLM faces numerous challenges in industrial environments characterized by constrained computation and communication (C^2^) resources, data privacy and security, and strong heterogeneity, as illustrated in Figure 4.

#### 4.1.1. C^2^ Overhead

During Fed-LLM training, computational resource overload on industrial devices becomes more pronounced compared to traditional ML. On resource-constrained industrial edge devices, such as programmable logic controllers (PLCs) and robot controllers, client-side training requires executing large-scale matrix operations, LoRA weight updates, or backpropagation computations. The peak computational power and memory consumption often exceed the capacity of most edge nodes, which can lead to increased inference latency, device overheating, and potential interference with existing real-time control logic.

Simultaneously, FL frameworks require many rounds of communication between clients and servers until the model converges. This communication usually consists of model parameters that have been updated locally [22]. However, LLM typically has billions of parameters (e.g., Gemma 7B, LLaMA2-7B). As a result, exchanging model parameters multiple times will add significant communication costs, which can cause issues such as transmission delays and saturate bandwidth [23].

Consequently, efficiently training a Fed-LLM under C^2^ constraints in industrial settings is still a central technology challenge for Fed-LLM deployment.

#### 4.1.2. Privacy and Security

While FL is broadly considered a distributed learning framework with privacy-preserving capabilities, real-world implementations in critical domains such as industrial manufacturing, energy, and power demonstrate a clear lack of privacy-protection inheritance during the aggregation stage [24,25]. If attackers conduct a man-in-the-middle attack on server communication channels or impersonate a valid aggregation node, they could recover sensitive local training data from only the gradients uploaded by clients. Such attacks would likely result in the leakage of valuable information [26,27]. Thus, maintaining safe aggregation while preserving model performance in complex industrial data flows is one of the fundamental challenges for the broad deployment of Fed-LLM.

#### 4.1.3. Heterogeneity

In Industry 4.0 contexts, federated clients such as production workshops, equipment terminals, and edge nodes, experience large differences in computational capabilities, network bandwidths, data distributions, and model architectures [28]. This multidimensional variability in federated clients causes performance imbalance, increased model bias, and slower global convergence in federated training of large models [29].

- Heterogeneity across devices leads to variation in computational power, storage, and communication ability at each node. This can lead to inconsistent local training times, with synchronization being penalized by low-resource nodes, leading to the common “straggler effect” [30].
- Heterogeneity in data, i.e., differences in the distribution, scale, and feature space of the data across nodes, leads to non-IID data. This issue ultimately results in the global model not being able to balance optimal solutions for all nodes, which leads to fluctuations in convergence and unequal model performance.
- Model heterogeneity refers to the discrepancies of model architectures, parameter scales, or module designs across clients. This makes traditional parameter aggregation methods like FedAvg ineffective while also complicating model fusion and knowledge transfer.

Heterogeneity challenges in Fed-LLM impact the whole Fed-LLM workflow from system design and training optimization to model aggregation and remain central concerns for their stable deployment in real-world applications. In the rest of this section, we review solution strategies for the aforementioned challenges.

### 4.2. Techniques for C^2^ Overhead

Current strategies for addressing computational and communication overhead issues can be broadly categorized into four types, including parameter-efficient fine-tuning (PEFT), sparsification and quantization, dynamic adjustment and adaptation, model partitioning and layered training. Table 4 provides an overview and classification of representative approaches addressing C^2^ Overhead challenges.

#### 4.2.1. PEFT

**Figure 5:**
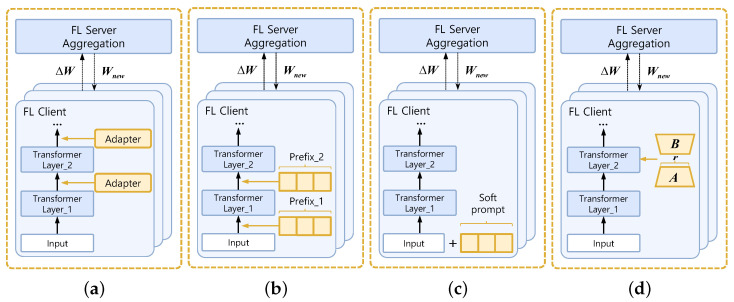
Representative PEFT methods integrated into federated learning frameworks: (**a**) adapter tuning; (**b**) prefix tuning; (**c**) prompt tuning; (**d**) Low-rank Adaptation (LoRA).

The fundamental principle of PEFT strategies is to freeze most model parameters while updating only a small number of modules, thereby transmitting only a fraction of parameters during federated training and communication [31]. As shown in Figure 5, early PEFT methods primarily employed structural insertion or input space adjustment patterns, with representative works including adapter tuning, prefix-tuning, and prompt-tuning [32].

**Adapter tuning** inserts lightweight knowledge modules between Transformer layers, updating only the parameters of these newly inserted modules while keeping the backbone network frozen to achieve task-specific fine-tuning [33]. This approach is widely adopted in FL to balance training efficiency and model performance [34]. FedAdapter proposes a federated version of the adapter module for federated fine-tuning in NLP scenarios, introducing a progressive adapter tuning strategy and adaptive parameter adjustment methods [35]. FedTT+ introduces the design of a tensorized adapter, representing the adapter module’s weights as tensor decompositions to reduce trainable parameters and communication volume on the client side [36].

**Prefix-tuning** methods append learnable prefix vectors before self-attention layers, concatenating them with input sequences for joint attention calculations [37]. This approach injects task-specific conditions into the model’s input space, focusing fine-tuning solely on prefix vector parameters. It has been extended for federated settings to reduce computational costs and parameter upload burdens. FedPrefix proposes training task-specific prefixes locally on clients while globally sharing the original attention layer to capture global dependencies. This enhances cross-task generalization while preserving privacy [38].

**Prompt tuning** represents a further lightweight, PEFT approach. It adds a set of learnable soft prompts only at the input layer [39]. These vectors are updated during backpropagation to guide the model toward performing specific downstream tasks [40]. Since only the prompt vectors are updated without altering the original model parameters, Prompt-tuning incurs significantly lower computational and communication costs than adapter and prefix architectures [41]. It is particularly well-suited for resource-constrained devices participating in training within federated environments. PromptFL distributes pre-trained base models (e.g., CLIP) to clients, which collaborate to optimize shared soft prompts using minimal local data without updating the model core [42]. FedPepTAO introduces partial prompt tuning to further reduce communication frequency, combining adaptive optimization algorithms to address data distribution heterogeneity and enhance the global model’s robustness and convergence efficiency [43].

Overall, these studies integrate PEFT concepts with FL frameworks, mitigating computational and communication burdens to some extent. However, significant limitations persist: adapter tuning increases model depth and reduces inference speed by inserting additional modules between layers; prefix tuning offers high compression rates but suffers from poor training stability, with fixed prefix lengths limiting contextual capacity; and prompt tuning requires the fewest parameters, but experiments demonstrate [44] that its performance degrades significantly in small-scale models or resource-constrained tasks. Its generalization capability depends on the scale of the pre-trained model, making stable transfer across heterogeneous federated clients challenging.

Compared to the aforementioned methods that explicitly modify model structures or input spaces, the emergence of **Low-Rank Adaptation (LoRA)** has introduced parameter low-rank reconstruction for PEFT [45]. The key assumption of LoRA is that the update matrix for pre-trained model weights exhibits low-rank properties during fine-tuning. Therefore, instead of altering the model’s original structure during training, LoRA introduces low-rank matrices into specific layers of the pre-trained model. Specifically, let the original weight matrix be $W_{0}\in R^{d\times k}$, the update term be ΔW, and the traditional fine-tuning update be $W=W_{0}+\Delta W$. LoRA decomposes ΔW into two low-rank matrices $A\in R^{d\times r}$ and $B\in R^{r\times k}$, as follows:

(2) ΔW=BA,r≪min(d,k)

where *r* is the rank constraint hyperparameter, typically set between 4 and 32. The forward propagation can then be expressed as

(3) $$h=W_{0}x+\alpha BAx$$

where α is the scaling factor controlling update magnitude. LoRA achieves model updates by training only the additional low-rank matrices *A* and *B*, decoupling from the original large model. This significantly reduces computational and transmission parameters while maintaining model performance.

LoRA has limited the communication load to tens of MB in Fed-LLM from hundreds of MB in an individual communication round [45]. This removes a significant burden on communication bandwidth, particularly within multi-node industrial edge devices or industrial edge-device setups. LoRA is also modular and can seamlessly integrate with different aggregation schemes such as FedAvg, FedProx and FedPer regardless of how differing the task is in terms of training and local adaptation. This is especially important in Industry 4.0 scenarios where differentiated knowledge can be fused from a variety of production lines, factories or devices.

The LoRA philosophy has spurred the emergence of multiple PEFT extensions based on low-rank structures in FL. These methods balance learning efficiency with model generalization for further optimization. Fed-IT combines instruction tuning with FL, enabling distributed collaborative instruction optimization for LLM by executing lightweight parameter updates like LoRA locally on each client [46]. FedSA-LoRA identifies that matrix A primarily encodes general knowledge while matrix B captures client-specific features [47]. Consequently, it uploads only matrix A to the server, significantly reducing communication overhead.

Table 5 summarizes the mentioned PEFT-based federated methods from the perspective of experimental setup, highlighting their implicit assumptions regarding federation scale, client capability, and backbone model size.

Adapter-based approaches are predominantly evaluated in cross-silo settings, where the client resources are relatively stable, allowing for additional architectural parameters to be maintained [35]. In contrast, prompt-based methods are typically adopted when task adaptation requires only shallow conditioning of pre-trained representations and client-side resources are highly constrained [38], which explains their frequent evaluation under cross-device settings with tens to hundreds of clients and medium-scale backbone models [43].

As the backbone model scales to billions of parameters (e.g., LLaMA-7B and LLaMA3-8B), LoRA-based approaches are more commonly employed [47], as low-rank weight adaptation better preserves model expressiveness while avoiding full-parameter updates, albeit at the cost of higher local computation compared to prompt tuning.

Taken together, these patterns indicate that different PEFT strategies are evaluated under distinct federated setups due to their differing trade-offs between scalability, computational overhead, and adaptation capacity, and that no single approach universally dominates across industrial deployment scenarios.

#### 4.2.2. Sparsification and Quantization

Sparsification and quantization strategies further apply sparsity or low-bit quantization to uploaded parameters, while incorporating PEFT modules such as LoRA and adapter, to minimize the number of bytes exchanged per communication.

FLASC employs Top-k magnitude sparse uploads for LoRA updates, coupled with indexing or compressed encoding to reduce communication [46]. RoLoRA combines LoRA with quantization robustness design through an alternating minimization strategy, addressing outlier and accuracy degradation issues under low-bit encoding while achieving only half the communication cost of LoRA [48]. FedPipe significantly reduces computational and memory overhead for local training on heterogeneous edge nodes through SVD-based adapter sparsification and adaptive quantization [49]. It further cuts communication load by aggregating only partial weights of lightweight adapters for synchronization.

A common advantage of these approaches is achieving communication compression ranging from several to tens of times while maintaining or only slightly sacrificing accuracy. However, engineering applications face challenges such as index overhead, cumulative quantization errors, and server-side denoising.

#### 4.2.3. Dynamic Adjustment and Adaptive

Dynamic adjustment and adaptive strategies optimize communication timing, client involvement, and aggregation methods to reduce unnecessary synchronization and improve bandwidth utilization or alleviate computational load on edge devices by scaling local updates.

SA-FedLoRA introduces a simulated annealing mechanism for dynamic parameter budget adjustment, reducing the number of parameters uploaded per round and mitigating client drift [50]. FlexLoRA enables dynamic adjustment of local LoRA rank, avoiding the requirement for all clients to synchronously use the same low-rank dimension [51]. During aggregation, heterogeneous LoRA ranks are weighted averaged, followed by weight redistribution via SVD.

Such methods are more beneficial in industrial environments with disparate devices and data; rich-resources nodes can take on heavier upload loads/updates that are more accurate, while weaker devices contribute sparse or quantized updates only when necessary. This overall improves the bandwidth efficiency while maintaining convergence properties.

#### 4.2.4. Model Splitting and Hierarchical Training

Model splitting and hierarchical training strategies include partitioning models across client devices, edge servers, or cloud platforms, when a single device is incapable of performing the task for the entire LLM. This is an effective means of alleviating computational and memory pressures on the endpoint, while also minimizing the number of parameters needed for synchronization.

SplitLoRA combines the advantages of FL and split learning (SL) [52]. Clients only perform forward propagation up to the split layer, uploading the resulting activations to the server, which then executes subsequent forward and backward propagation. Fed-piLot models the computational power, memory, and bandwidth differences across clients, employing an optimization strategy to assign differently sized LoRA modules to each client [53]. FedRA achieves heterogeneous-aware fine-tuning through randomized LoRA allocation without explicit device capability modeling, significantly reducing computational load on weaker devices while simultaneously compressing communication overhead per round [54].

The advantage of such approaches is enabling participation of extremely constrained devices in Fed-LLM fine-tuning, while the drawbacks include handling communication delays caused by splitting, privacy risks from activation leakage, and cross-layer synchronization complexity.

Beyond these mainstream fine-tuning approaches, additional studies leverage zero-order optimization, model compression, model pruning, and distributed learning to balance training resource consumption and performance. FedKSeed employs zero-order optimization using a finite set of random seeds, achieving efficient fine-tuning with only a few seeds and scalar gradients [55]. FedSpaLLM effectively reduces uploaded parameter size through personalized pruning based on client resources [56]. FedBiOT compresses LLM on the server while allowing clients to fine-tune only lightweight adaptation layers [57]. Dec-LoRA employs a decentralized federated fine-tuning architecture using LoRA to fine-tune LLM without relying on a central server, offering potential support for more robust and scalable federated fine-tuning in industrial network topologies [58].

#### 4.2.5. Conclusion of C^2^ Overhead Techniques

Existing C^2^ reduction strategies for Fed-LLM target different system bottlenecks, including communication volume, synchronization frequency, and client-side computation capacity. Those strategies are largely complementary rather than competing. Consequently, practical industrial deployments typically require a coordinated combination of PEFT, compression, and adaptive system strategies, instead of relying on a single optimization technique, to achieve scalable and robust performance.

### 4.3. Techniques for Privacy and Security

Mainstream privacy protection techniques for Fed-LLM include: differential privacy (DP), homomorphic encryption (HE), and secure multi-party computation (SMPC).

#### 4.3.1. DP

DP balances privacy protection and model performance by introducing noise into model parameters or gradients before uploading [59]. Its core definition states that a random algorithm M satisfies (ϵ,δ)-DP when any two adjacent datasets *D* and $D^{\prime }$ differ by at most one sample, provided that for any output set *S*:

(4) $$\mathrm{Pr}\left[M\left(D\right)\in S\right]\leq e^{\epsilon }\mathrm{Pr}\left[M\left(D^{\prime }\right)\in S\right]+\delta$$

where ϵ controls the privacy budget (smaller values indicate stronger privacy) and δ represents the tolerated leakage probability. In federated training, DP is typically implemented through gradient perturbation or output perturbation. The core idea of the typical algorithm DP-FedAvg is to clip and inject Gaussian noise into the gradient $g_{i}$ uploaded by each client before each communication round [42]:

(5) $${\tilde{g}}_{i}=\mathrm{Clip}\left(g_{i},C\right)+N\left(0,\sigma^{2}C^{2}I\right)$$

Here, *C* denotes the gradient clipping threshold, and σ represents the noise intensity. This mechanism prevents malicious aggregators or other clients from reconstructing original data through gradient back-analysis. Methods like DP-LoRA combine DP with PEFT, adding noise only to low-rank adapters to balance privacy and utility [60].

#### 4.3.2. HE

HE enables direct addition or multiplication within ciphertext space, facilitating model aggregation or gradient updates without decryption [61]. For Fed-LLM, HE allows servers to perform weighted averaging without viewing plaintext gradients, ensuring end-to-end encryption during parameter transmission and aggregation. An encryption scheme Enc() qualifies if:

(6) Enc(a)⊕Enc(b)=Enc(a+b)

or

(7) Enc(a)⊗Enc(b)=Enc(a×b)

it is termed additive or multiplicative homomorphic. Fully homomorphic encryption (FHE) schemes like BFV and CKKS support both operations, while partially homomorphic encryption (PHE) schemes such as Paillier and RSA support only additive or multiplicative operations.

Due to the increased gradient dimension after HE encryption leading to high computational complexity and communication overhead, lightweight approximate homomorphic schemes have emerged as a research focus in recent years. The FedML-HE model employs lightweight HE at both encryption and aggregation ends, encrypting only privacy-sensitive module parameters for transmission [62], thereby balancing performance and security.

#### 4.3.3. SMPC

SMPC evolved from Yao’s secure two-party computation [63], enabling multiple clients to collaboratively perform computational tasks like model aggregation without revealing their inputs. A conventional secure aggregation algorithm can be formulated as follows, given *n* clients with local model gradients $g_{1},g_{2},\ldots ,g_{n}$, the objective is to compute:

(8) $$G=\sum_{i=1}^{n}g_{i}$$

while the aggregation server must not learn any individual $g_{i}$ values. The SecAgg algorithm proposed by Bonawitz et al. combines random masking with a key-sharing mechanism [64]. Each client generates a random vector $r_{ij}$ and exchanges it with other clients, ensuring that:

(9) $$g_{i}^{\prime }=g_{i}+\sum_{j\neq i}\left(r_{ij}-r_{ji}\right)$$

After aggregation, all masks cancel each other out, leaving only the true global gradient. In Fed-LLM, SMPC is primarily used for secure aggregation and distributed parameter updates. ELSA leverages SMPC principles by enabling clients to act as “untrusted dealers” for generating and distributing masked shares [40]. This avoids complex server-to-server interactions, prevents any single malicious participant from accessing other clients’ gradients, and ensures both the correctness and privacy of aggregated results.

#### 4.3.4. Comparison of Privacy and Security Techniques

As shown in Table 6, we summarized the trade-offs between different privacy-preserving techniques across dimensions like privacy-level, accuracy impact, C^2^ cost, and scalability.

Although DP is lightweight and scalable, introduces stochastic noise that may degrade convergence stability and prediction accuracy, particularly in industrial tasks requiring high precision and temporal consistency. HE preserves model accuracy by operating on encrypted updates, but the resulting high-dimensional ciphertexts significantly increase computation latency and communication overhead, rendering it impractical for large-scale industrial models with billions of parameters. SMPC provides strong security through collaborative protocols. However, its multiple interaction rounds and protocol complexity hinder efficient execution on resource-constrained edge devices.

From a deployability perspective, DP-based approaches are more suitable for large-scale, cross-device industrial scenarios where scalability and low overhead are prioritized over strict accuracy guarantees. In contrast, HE and SMPC are better aligned with cross-silo or small-scale industrial settings involving limited participants and stringent privacy requirements, such as inter-organizational collaboration or sensitive industrial data sharing. These comparisons highlight that no single privacy mechanism universally dominates. Instead, algorithm selection must be guided by the specific industrial deployment context.

### 4.4. Techniques for Heterogeneity

To address heterogeneity challenges, FL approaches typically require adaptive adjustments at the device, data, and model levels [65]. Heterogeneous FL must balance personalized optimization with improved global generalization performance while ensuring global collaboration [66], accounting for differences in computational resources, data distribution, and model structures across clients. Table 7 provides an overview and classification of representative approaches addressing heterogeneity challenges.

#### 4.4.1. Device Heterogeneity

In industrial scenarios, device heterogeneity often manifests as weak devices being unable to support full-model training, differences in local training durations, and frequent communication. Approaches to address these issues include PEFT, model decomposition with partial training, and asynchronous aggregation in FL. The discussion of computational and communication overload above has already covered how PEFT and model decomposition with partial training can mitigate resource consumption and bandwidth constraints.

Asynchronous aggregation allows clients to submit updates at their own pace, without requiring the server to wait for all clients to complete a training round. This reduces bottlenecks caused by low-resource nodes. FedASMU adopts staleness-aware aggregation, where the server assigns different weights based on the timeliness of updates, giving more importance to recent contributions [67]. FedVS ignores delayed updates from certain clients and reconstructs the global embeddings losslessly using the contributions from the remaining clients [68]. The asynchronous mechanism can effectively improve system throughput and training efficiency, which is particularly important for industrial deployments with large-scale and heterogeneous resources.

#### 4.4.2. Data Heterogeneity

In Fed-LLM, data heterogeneity often slows global model convergence or even causes oscillations. It can also degrade performance for individual clients and make it difficult to balance global model generalization with client-specific personalization. Relevant studies have explored FL approaches based on regularization, adaptive aggregation, client clustering, meta-learning, and multi-task learning [69].

**Regularization** approaches prevent local overfitting by introducing global model constraints or parameter similarity regularization terms into local optimization objectives. Early work like FedProx improved upon FedAvg by incorporating regularization terms into client-side training loss functions:

(10) $$F_{i}^{\mathrm{prox}}\left(w_{i}\right)=F_{i}\left(w_{i}\right)+\frac{\mu }{2}\left|\right|w_{i}-w_{g}{\left|\right|}^{2}$$

to stabilize the local optimization process under heterogeneous data and constrain the divergence between local and global models [70]. Fed-ET combines weighted consensus distillation with diversity regularization. The method ensures the reliability of the consensus reached by the ensemble, while enhancing model generalization by leveraging diverse datasets [71].

**Adaptive aggregation** approaches introduce adaptive weighting during federated aggregation to mitigate the averaging problem caused by heterogeneous client data. Each client’s contribution to the global model depends on its data characteristics, model performance, or gradient similarity.

FedAdam incorporates momentum and adaptive learning rate adjustment on the server to smooth differences between client-uploaded gradients [72]. Specifically, after several rounds of local SGD updates, clients upload the weight update differences to the server. The aggregated global gradient on the server can be expressed as $g_{t}$. An adaptive momentum mechanism is then introduced based on this:

(11) $$\begin{array}{cc} \; & m_{t}=\beta_{1}m_{t-1}+\left(1-\beta_{1}\right)g_{t}\\ \; & v_{t}=\beta_{2}v_{t-1}+\left(1-\beta_{2}\right)g_{t}^{2}\\ \; & w_{t+1}=w_{t}-\eta \frac{m_{t}}{\sqrt{v_{t}}+\epsilon } \end{array}$$

where $m_{t}$ and $v_{t}$ denote first- and second-order moment estimates, $\beta_{1}$ and $\beta_{2}$ are smoothing coefficients, and η is the learning rate. PFPT maintains a probability distribution over prompts on each client instead of deterministic vectors, enabling collaborative optimization across clients [73]. With uncertainty incorporated through global aggregation, PFPT enhances generalization and robustness of the model under heterogeneous data. In industrial settings where there is a strong shift in data and dynamic changes in the tasks, such adaptive learning techniques help to ensure that models can have relatively invariant learning with respect to the operating conditions.

**Client clustering** approaches address scenarios where client data exhibit group-wise similarity. Clients within the same cluster share model parameters, while different clusters are optimized independently, forming multiple sub-global models. IFCA maintains multiple global models on the server [74]. In each training round, clients select the most suitable global model for local updates based on their data distribution and model performance, then upload updates to the corresponding model group. CASA further considers practical factors such as asynchronous client arrivals and communication delays, introducing a caching mechanism to correct similarity calculations [75]. Clustering strategies improve the model’s ability to capture local patterns and provide a structural basis for subsequent model selection and knowledge transfer.

**Meta-learning and multi-task learning** approaches are also important for addressing data heterogeneity. Meta-learning aims to learn a strategy from the global task distribution that can quickly adapt to different client tasks [71]. Per-FedAvg learns a set of global initialization parameters on the server, allowing clients to converge to optimal local models with few local updates [76]. **Multi-task learning** treats client tasks as related but non-identical objectives. By introducing task relationship matrices or parameter-sharing structures at the global level, it balances transfer and isolation between tasks. Some studies incorporate task-weight adaptation within the federated framework, enabling effective modeling of inter-client relationships even under highly non-IID data.

#### 4.4.3. Model Heterogeneity

Model heterogeneity arises from differences in client-deployed models or inconsistencies in parameter dimensions or gradient spaces due to varying optimization objectives [77]. Relevant studies include FL approaches based on knowledge transfer and subnetwork extraction.

**Knowledge transfer** approaches align models in a structure-agnostic manner by exchanging knowledge between the global server model and client models through soft labels or intermediate representations. FedMKT performs bidirectional knowledge distillation (KD) between large and small models by exchanging logits (soft labels), requiring only output dimension consistency rather than structural matching [78]. FEDSP retains local audio features and model updates on the client while uploading only encrypted or distilled representations [79]. FedTED addresses heterogeneity with a dual-branch predictor and data-free distillation, where clients upload only the general branch, and the server synthesizes public features using an adversarial generator to distill a global feature extractor [80]. Fed-ET allows clients to train heterogeneous small models locally and applies weighted consensus distillation with diversity regularization on ensemble outputs at the server, enabling large model training without model homogeneity [71]. FedGKT combines gradient distillation with layer-wise partitioning, allowing low-resource clients to retain only partial trainable layers while offloading remaining layers to the cloud for joint optimization, aligning models via gradient space despite structural differences [81].

**Subnetwork extraction** divides the global model into multiple independently trainable or activatable subnetworks, supporting deployment under varying resource conditions. RaFFM performs importance-based parameter extraction and subnetwork compression on transformer-based base models, enabling resource-aware distributed submodel deployment [82]. This approach allows base models to adapt to heterogeneous devices in federated environments, significantly reducing resource consumption while maintaining performance, providing a practical solution for deploying large models in real-world edge FL systems.

#### 4.4.4. Conclusions of Heterogeneity Techniques

Device heterogeneity is addressed by PEFT, partial model training, and asynchronous aggregation, which reduce computation and communication bottlenecks for weak or slow clients [67]. Industrial deployments typically benefit from controlled asynchrony, balancing efficiency, scalability, and convergence despite diverse device capabilities.

Existing approaches to data heterogeneity in Fed-LLM can be viewed as operating at different levels of intervention. Regularization and adaptive aggregation offering simplicity and robustness but limited personalization [71]. In contrast, client clustering and meta-/multi-task achieving better local adaptation at the cost of higher system complexity and coordination overhead [71,74]. Consequently, industrial Fed-LLM systems often combine lightweight stabilization mechanisms with selective personalization strategies to balance convergence efficiency and client-level performance under highly non-IID data.

In addressing model heterogeneity, knowledge transfer methods enabling heterogeneous models to participate [71], but often incur additional training or communication overhead. Subnetwork extraction, by contrast, directly adapts the global model to device capabilities through structured sparsification or modular activation [82], offering better efficiency and deployment simplicity. As a result, knowledge transfer is more suitable for cross-model collaboration, while subnetwork-based methods are better aligned with large-scale, resource-diverse industrial Fed-LLM deployments.

While these methods have made remarkable advancement in mitigating major Fed-LLM bottlenecks, most are simply local improvements and may not be optimized together for multiple objectives. Furthermore, challenges are exacerbated in Industry 4.0 settings, which demand high reliability, real-time capabilities, and strict security limitations. Thus, finding an effective trade-off between communication efficiency, privacy protection, and multi-source heterogeneity continues to be a fundamental research issue for implementing Fed-LLM in complex and dynamic industrial production settings.

## 5. LLM and FL Synergies for Industry 4.0

Thanks to the promotion of Industry 4.0, LLMs have started to emerge in areas such as industrial text understanding and product lifecycle management (PLM). FL has shown efficient data privacy protection in scenarios such as PdM, QC, and SCM again.

Yet, the joint use of LLM and FL in real industrial infrastructures, while instrumental in demonstrating readiness, is still at an embryonic stage of exploration, with most studies still stuck in lab studies or simulated environments. As we explore this evidence, we must acknowledge that fairly heterogeneous devices, including our robot controllers, production line terminals, and quality inspection systems, must adhere to the strict industrial requirements for computing capability, network connectivity, and reliability, which pose considerable challenges when attempting an end-to-end deployment of the concept Fed-LLM. This section reviews the relevant progress elsewhere.

### 5.1. LLM-Empowered Industry 4.0

As Industry 4.0 transitions from automation to intelligence, LLMs are increasingly assumed to be a semantic engine linking manufacturing knowledge, production processes, and intelligent services. More fundamentally, LLMs should not be defined by their NLP capabilities; LLMs are capable of effective semantic modeling and contextual reasoning and can build a common knowledge representation layer for an industrial context. That includes leveling up the intelligence in design, manufacturing, and operations.

Industrial know-how often exists in poorly arranged, relative knowledge spread across a variety of technical documents plus process procedures, maintenance records, and equipment instructions. LLM, with its high-fidelity semantic parsing, can turn disparate these textual insights into structured, inferable symbols of manufacturing knowledge. Specifically in industrial machinery faults diagnosis situations, LLM can automatically correlate “fault patterns-analysis-remediation actions” from annual relic maintenance records, contributing high-fidelity structured knowledge inputs into a fault diagnosis model [83]. In chemical process companies, LLMs read both operational manuals and also risk handbooks to suggest operational guidance using question-answering, significantly reducing both the retrieval of knowledge and transfer of experience costs [84].

**Task Assistance in Manufacturing Processes:** LLMs exhibit generalization abilities across modalities and processes, especially when considering tasks related to the initial design and process planning stages. They are capable of automatically generating engineering requirement specifications from a functional description, yielding early-stage design concepts, 3D models, or recommendations for manufacturability analysis [10]. Multiple studies demonstrate that LLM can convert textual requirements into CAD scripts, parametric sketches, or 3D models [85,86,87]. They can even be embedded throughout the entire design-to-manufacturing workflow to generate manufacturing instructions and evaluate design performance [88].

**Intelligent Decision-Making and Operations Support:** By integrating data from different departments and systems, such as MES, ERP, and SCM [89], LLM performs high-level semantic reasoning to produce actionable decisions for production management and maintenance. In operations, RAG-enhanced LLM leverage time-series sensor and operational log data from plastic manufacturing plants and SKAB benchmarks to provide real-time anomaly detection and decision support [90]. Furthermore, combining vibration monitoring data with RAG-retrieved equipment knowledge allows LLM to implement a complete intelligent operations workflow from anomaly detection to executable maintenance plan generation, significantly improving the automation of predictive maintenance [91].

Current industrial-grade LLM applications still primarily rely on centralized training and private deployment, resulting in long model update cycles, limited generalization capabilities, and insufficient cross-facility transferability. As industrial data continue to grow, enabling LLM to collaboratively participate in these industrial processes while ensuring data security and privacy remains a critical challenge for advancing LLM applications in Industry 4.0.

### 5.2. FL-Empowered Industry 4.0

In Industry 4.0, ongoing research has explored embedding FL into multiple scenarios such as smart manufacturing, SCM and logistics management, and operations management, to balance data privacy, security, and model generalization [7].

**Smart Manufacturing:** FL enables collaborative training of ML models across different entities to support QC, improve productivity and product quality, and reduce costs. SecFL predicts sheet metal forming defects under variable manufacturing conditions, reducing automobile scrap rates [92]. In the aerospace sector, FL is used to train cross-enterprise predictive models for planning processing parameters of aircraft structural components [93]. Using FedAvg and YOLOv5 for object detection, quality inspection models were trained collaboratively across multiple factories. The federated models demonstrated better generalization across all participants than models trained locally in a single factory, effectively reducing dependence on individual factory biases [94].

**SCM and Logistics Management:** FL enables supply chain risk prediction and logistics efficiency optimization. A predictive model trained using FL and data from multiple factories of varying scales was applied to forecast factory delivery delays. Experiments demonstrated FL outperforms centralized training or locally learned models, particularly benefiting participants with limited data [95]. Furthermore, FL trained fault prediction models while preserving AGV (automated guided vehicle) local data privacy. Compared to traditional centralized training methods, it demonstrated superior performance in predicting transient energy consumption signal anomalies. After completing eight rounds of federated aggregation, the global model’s predictive performance improved by approximately 19%, enhancing vehicle availability and logistics efficiency [96].

**Operations and Maintenance Management:** In operations management, FL is primarily applied to fault detection and PdM. For fault detection, cloud–edge collaborative diagnostic frameworks based on FL can significantly improve fault recognition accuracy and generalization without sharing raw turbine data, thereby enhancing wind power operations efficiency and reliability [97]. For PdM, multiple airlines have collaboratively trained engine remaining useful life prediction models using FL. This approach achieves high-precision predictions without sharing raw engine sensor data, substantially improving model generalization across fleets and operational conditions, demonstrating the feasibility of FL for high-value asset lifecycle management [98]. Considering variations in the distribution of time-series data collected from equipment, the FL-based 1DCNN-BiLSTM model can be applied to time-series anomaly detection and PdM in manufacturing processes [99]. Through collaborative training across multiple clients, it achieves high-precision anomaly detection with an accuracy of 97.2%.

In summary, FL enables collaborative modeling with invisible data across multiple typical industrial scenarios, making cross-enterprise and cross-device model construction feasible. However, FL still faces two major limitations in industrial tasks:

- First, conventional FL is mainly applied to small-to-medium-sized models, with limited support for complex semantic reasoning tasks.
- Second, data heterogeneity, device heterogeneity, and strict compliance requirements in industrial scenarios continue to challenge model robustness, security, and scalability. With growing demand for stronger semantic understanding in industrial systems, integrating LLM into FL has become a natural progression.

### 5.3. Fed-LLM-Empowered Industry 4.0

Fed-LLM signifies a new domain of research in recent years with the aim to involve LLM in collaborative training and updates across nodes of heterogeneous levels in industrial systems while remaining private and compliant. The Fed-LLM approach aspires to deliver a more powerful unified semantic reasoning hub for industry applications.

#### 5.3.1. Open Fed-LLM System

Fed-LLM systems form the critical foundation for advancing LLM deployment and implementation in industrial domains. Early FL systems (e.g., TensorFlow Federated and PySyft) primarily focused on parameter aggregation and privacy protection for small-scale neural networks and were typically released as open-source frameworks to support research and prototyping. However, when confronting LLMs characterized by massive parameters, complex architectures, and high communication demands, these systems struggle to meet the requirements for performance, scalability, and security mechanisms.

With advancements in PEFT, KD, and model compression, academia and industry began exploring how to achieve federated training and collaborative optimization of large models under data silos, heterogeneous devices, and strict privacy and security requirements. Consequently, a series of Fed-LLM systems emerged.

As shown in Table 8, we summarized systems developed and released in open-source form to enable reproducibility and practical engineering validation. These systems are primarily developed and maintained by academic institutions or industry–academia collaborations, aiming to promote reproducibility, extensibility, and practical adoption in real-world industrial scenarios. This openness significantly lowers the barrier for deployment, customization, and secondary development, which is particularly valuable for industrial engineers and system developers.

**FATE-LLM** constructs a FL framework for industrial-grade LLM, aiming to address data security and model intellectual property protection in highly regulated industries such as finance and healthcare [100]. By integrating model-level DP and HE, FATE-LLM enables secure federated training of heterogeneous large models. It utilizes SMPC and verifiable aggregation methods to assure verifiable contributions so maintain the privacy of participants. FATE-LLM offers a measurable security and compliance mechanism for multi-institutional collaborative training exercises in an industrial environment while balancing protection of the model, privacy of the data, and coordination of knowledge across multiple nodes and institutions.

**FederatedScope-LLM (FS-LLM)** constructs an end-to-end Fed-LLM fine-tuning benchmark system, supporting rapid deployment and performance comparison of mainstream PEFT methods such as adapter tuning, LoRA, and prompt tuning in federated environments [101]. FS-LLM particularly emphasizes model personalization and task transferability, allowing clients to maintain local model characteristics while sharing global knowledge. This approach balances model customization and generalization performance in industrial applications. Notably, the system incorporates a standardized evaluation workflow, providing a benchmark for comparative experiments and algorithmic reproducibility of industrial multi-task models.

**Shepherd** is a slim federated instruction fine-tuning framework for large models. Clients produce updated model adapters based on local instructions, while the server is only responsible for scheduling tasks and overall model aggregation. Shepherd covers the main processes of client data allocation, training scheduling, simulating local updates, and model aggregation—all in a way that can increase the efficiency of federated large language model training at the system level [46].

**OpenFedLLM** is the first framework to concurrently introduce federated command tuning, federated value alignment techniques, and diversified FL baselines, improving the consistency of collaborative reasoning of large models across distributed environments [102]. The system includes multiple classic FL algorithms, such as FedAvg, FedProx, and SCAFFOLD, combined with LoRA PEFT strategies and quantization communication compression techniques to improve performance. A diversified set of domain training datasets is also offered, as well as rich support for evaluation metrics.

In terms of the feasibility of industrial deployment, FATE-LLM supports industrial-scale deployment on multi-GPU clusters, using PEFT to reduce trainable parameters to 0.01%∼0.1%, lowering communication overhead [100]. Combined with FATE-Flow for task orchestration and optional KubeFATE integration, it maintains operational stability in homogeneous or heterogeneous cluster environments. Shepherd relies primarily on underlying framework checkpointing for robustness [46]. FS-LLM and OpenFedLLM integrates multiple FL algorithms for benchmarking, but lacks explicit fault recovery and asynchronous straggler mitigation [101], does not offer industrial-grade resource orchestration or stability guarantee [102].

Overall, while FS-LLM, OpenFedLLM, and Shepherd are primarily designed for research and algorithmic validation, FATE-LLM demonstrates stronger industrial readiness by jointly addressing scalability, communication efficiency, and system-level robustness.

#### 5.3.2. Fed-LLM-Empowered Industry 4.0

In industrial settings, Fed-LLM integrates the capabilities of LLM and FL, making its potential applications particularly valuable:

- First, it ensures sensitive process documentation, quality records, and production log privacy data remain within the domain at the equipment level.
- Second, it enhances the model’s generalization capabilities for anomaly patterns, process semantics, and multi-device collaboration strategies by aggregating cross-factory domain knowledge.
- Third, it supports continuous learning and scenario adaptation for LLM in highly dynamic manufacturing workshops with frequently changing tasks, reducing manual maintenance costs.

Existing verifiable Fed-LLM applications primarily focus on hybrid FL architectures, such as partitioning LLM models between edge and cloud layers. Clients adapt locally based on their specific production line conditions, delegating global model updates to centralized cloud nodes [103]. Addressing the challenges of resource-constrained industrial edge devices like PLCs and industrial gateways with stringent real-time requirements, the LLMCAD employs hierarchical computation and low-bit quantization (4-bit or lower) alongside customized kernels [104]. This approach minimizes memory footprint and computational complexity while preserving inference accuracy, accelerating LLM reasoning by several orders of magnitude. It provides the technical foundation for sub-second real-time fault diagnosis and local federated fine-tuning on IIoT devices.

FCLLM-DT addresses challenges in IIoT fault diagnosis—such as sensor data anomalies, data interruptions, and cross-factory privacy risks—by integrating digital twins for data repair and RAG-enhanced LLM for virtual data generation in sensor failure scenarios [105]. It achieves privacy-preserving collaborative training of multi-factory models through federated continuous learning. Performance validation on simulated environments and publicly released industrial fault diagnosis datasets demonstrates that the framework outperforms alternative approaches in both sensor data quality restoration and bearing fault diagnosis accuracy.

Overall, Fed-LLMs represent the future trend of integrating semantic intelligence with privacy-preserving computing in Industry 4.0. However, their large-scale deployment requires breakthroughs across multiple dimensions, including architecture, algorithms, systems engineering, and adaptation to industrial protocols.

## 6. Challenges and Future Directions

While Fed-LLM shows a great potential in Industry 4.0, the implementation challenges to scale it presents systemic issues. These challenges span computation, communication infrastructure, and industrial protocols. Future research directions may evolve from technological and engineering perspectives.

### 6.1. Industrial-Grade Lightweighting

While industrial edge nodes embedded GPUs with VRAM lower than 16 GB, conventional lightweighting approaches, such as model pruning and quantization, are insufficient to really comprehend the very limited computational capacity at industrial edge nodes for LLM with billions of parameters.

In particular, attention needs to be directed toward edge-computing-aware adaptive PEFT, which means not simply adapting the rank of LoRA or adapters dynamically but also investigating federated adaptive expert selection mechanisms, enabling edge nodes with differing processing capabilities to activate and update only the subsets of experts most relevant to their local task, thus enabling efficient on-demand computation and federated sparse aggregation [106]. LoftQ [107] provides a quantization-aware initialization that mitigates the discretization error of low-rank approximations, thereby drastically reducing VRAM overhead for industrial nodes, particularly in 2-bit and 4-bit regimes. FRLoRA [108] advocate for a more granular optimization of the 0.01%∼0.1% parameter budget by selectively updating both low-rank matrices and the backbone, ensuring that federated sparse aggregation does not compromise the global model’s reasoning integrity.

### 6.2. Industrial Deep Heterogeneity

In industrial contexts, there is heterogeneity that goes well beyond non-IID statistical challenges, where fundamental differences exist due to underlying semantics and knowledge systems varying from factory to factory and in the generations of equipment, for example, different failure mechanisms associated with particular processes.

Consider integrating federated cross-domain semantic alignment with distillation techniques, extract generalizable knowledge representations (e.g., physical laws) across industrial environments [109], ensuring the global model captures cross-enterprise universal mechanisms. FedSKD [110] demonstrates that multi-dimensional similarity-based distillation can enable effective knowledge transfer across fully heterogeneous client models without relying on parameter aggregation. FedProtoKD [111] explicitly align class-level representations across heterogeneous participants. End-edge–cloud federated learning studies show that self-rectified knowledge agglomeration can reduce semantic drift across layers [112], pointing to a potential research direction in aligning multi-level industrial knowledge systems.

### 6.3. RAG-Enhanced Industrial Fed-LLM

In industrial environments, knowledge relevant to decision-making is often distributed across heterogeneous and continuously evolving data sources, such as equipment manuals, maintenance logs, process specifications, and operational databases. Purely parametric Fed-LLMs face inherent limitations in capturing such rapidly changing and domain-specific knowledge, especially when direct data sharing across enterprises is restricted by privacy and regulatory constraints.

A promising research direction is the integration of retrieval-augmented generation (RAG) mechanisms into federated LLM frameworks, enabling models to access external, non-parametric industrial knowledge while preserving data locality. Recent studies on federated RAG indicate that decoupling knowledge storage from model parameters can substantially reduce catastrophic forgetting and communication overhead [113], while improving adaptability to domain shifts. For instance, federated RAG frameworks demonstrate that retrieval modules can be collaboratively optimized without exposing raw documents, allowing global models to leverage cross-enterprise knowledge through privacy-preserving retrieval interfaces [113]. Such approaches suggest a potential pathway for industrial Fed-LLMs to dynamically incorporate evolving operational knowledge while maintaining robustness under heterogeneous data ownership and access policies [114].

### 6.4. Machine Unlearning and Continual Learning for Fed-LLM

Industrial Fed-LLMs are increasingly required to operate over long lifecycles, during which data distributions, operational conditions, and regulatory requirements continuously evolve. Continual learning is therefore essential to incrementally integrate new knowledge without degrading previously acquired capabilities. However, in federated settings, continual adaptation must be achieved under strict resource constraints and without centralized access to historical data.

Recent work such as SacFL explicitly addresses this challenge by proposing self-adaptive federated continual learning mechanisms tailored for resource-constrained end devices, demonstrating that selective parameter adaptation can mitigate catastrophic forgetting while maintaining communication efficiency [115]. Complementarily, regulatory compliance introduces the additional requirement of machine unlearning, where specific data contributions must be removed upon request. The study Unlearning through Knowledge Overwriting shows that reversible federated unlearning can be achieved via selective sparse adapters, enabling targeted knowledge removal without full retraining [116].

## 7. Conclusions

This review systematically examines the integration of LLMs and FL within Industry 4.0, covering architectural foundations, enabling techniques, and representative application scenarios. While our analysis incorporates recent advances from both academic and industrial perspectives, it should be noted that the Fed-LLM landscape is evolving rapidly, and new methods may emerge beyond the temporal scope of this survey. Moreover, by focusing on the integration of LLMs, FL, and Industry 4.0, certain adjacent topics—such as standalone edge intelligence or centralized industrial foundation models [117]—may not have been discussed in equal depth. These factors should be considered when interpreting the conclusions drawn in this work.

A key conclusion of this work is that Fed-LLMs hold strong potential for building collaborative, privacy-preserving industrial intelligence, yet their practical deployment faces fundamental challenges that cannot be addressed by directly transplanting existing LLM or FL techniques.

Specifically, the challenges identified in this review point to several open research directions. First, the severe mismatch between the resource constraints of industrial edge nodes and the scale of modern LLMs calls for industrial-grade lightweighting strategies that go beyond static compression, emphasizing adaptive and federated parameter-efficient mechanisms [106]. Second, industrial environments exhibit deep heterogeneity in semantics and knowledge structures [109], requiring new FL paradigms capable of aligning cross-domain industrial knowledge [110,111]. Third, the reliance on rapidly evolving and distributed industrial knowledge sources motivates the integration of retrieval-augmented generation into federated frameworks [113,114], enabling dynamic knowledge access without violating data sovereignty. Finally, the long lifecycle of industrial systems introduces the dual requirements of continual learning and machine unlearning [115,116], raising fundamental questions about how Fed-LLMs can adapt over time while remaining efficient, compliant, and trustworthy.

Overall, there remains a substantial gap between current research prototypes and reliable large-scale deployment. Bridging this gap will require coordinated advances in algorithms, systems, and evaluation methodologies. By synthesizing recent progress and systematically articulating these open challenges, this review aims to provide a forward-looking perspective to guide future research on Fed-LLM for Industry 4.0.

## Figures and Tables

**Table 1:** Comparison to related reviews. The symbols ✔ and ✘ denote the presence or absence of the corresponding topic in each review.

Review	Industry 4.0	LLM	Data Soils	Key Contribution	Differences from Our Review
Chkirbene et al. [3]	✔	✔	✘	Discusses the technological evolution of LLM and investigates their practical applications in automation, decision-making, and content generation across industries (e.g., healthcare, finance, and customer service).	This work focuses on the application of general-purpose LLM in specific industries, lacking detailed research on privacy protection issues in industrial scenarios.
Raza et al. [4]	✔	✔	✘	The system summarizes the development, architecture, and industry applications of LLM, while addressing security, privacy, and ethical concerns.	This work focuses on the applications and challenges of LLM across multiple industries and does not specifically explore introducing FL to address privacy concerns.
Cheng et al. [5]	✘	✔	✔	Summarizes key technologies and applications of LLM and FL, presenting motivations and challenges; discusses privacy issues in Fed-LLM.	This work focuses on the combination of FL and LLM. Our review emphasizes discussions on Fed-LLM in industrial settings.
Syed et al. [6]	✔	✘	✔	Systematically summarizes the application of FL in addressing issues related to data privacy, data security, and anomaly detection within Industry 4.0 manufacturing environments.	This work focuses on FL applications in Industry 4.0 manufacturing. Our review primarily discusses the integration of LLM with FL to address data silos and privacy challenges in industry.
Leng et al. [7]	✔	✘	✔	Considering privacy and security requirements in manufacturing, this work discusses the application of FL in intelligent manufacturing systems and product lifecycle management (PLM).	This work focuses on the application of FL in specific manufacturing domains. Our review primarily discusses the integration of LLM with FL to address data silos and privacy-related issues in industrial settings.
Yang et al. [8]	Partial	✔	✔	Discusses the synergistic application of IoT, LLM, and FL in edge computing environments to address privacy protection challenges.	This work focuses on the integration of IoT, LLM, and FL, whereas our review encompasses a broader range of industrial application scenarios.
Our review	✔	✔	✔	Exploring the synergistic mechanisms, typical applications, and key challenges of integrating LLM with FL in Industry 4.0.	—

**Table 2:** Search Strings and Databases. The table summarizes the databases and corresponding search strings used in this review. In addition to the core keywords, challenge-related terms were also included to capture studies addressing practical deployment and optimization issues. For the Fed-LLM query, these challenge-related terms were intentionally incorporated to constrain the retrieval scope and prioritize studies with direct relevance to real-world system considerations.

Database	Search String			
	FL and LLM	FL in Industry	LLM in Industry	Fed-LLM in Industry
Web of Science/IEEE Xplore/ACM/Scopus	(“federated learning” OR FL) AND (“large language model” OR LLM) AND (“resource constrained” OR “communication” OR “aggregation security” OR “heterogeneity”)	(“federated learning” OR FL) AND (“Industry 4.0” OR “manufacturing”)	(“large language model” OR LLM) AND (“Industry 4.0” OR “manufacturing”)	(“federated learning” OR FL) AND (“large language model” OR LLM) AND (“Industry 4.0” OR “manufacturing”)
arXiv	federated learning AND large language model AND resource constrained; federated learning AND large language model AND communication; federated learning AND large language model AND aggregation security; federated learning AND large language model AND heterogeneity	federated learning AND Industry 4.0; federated learning AND manufacturing	large language model AND Industry 4.0; large language model AND manufacturing	federated learning AND large language model AND Industry 4.0; federated learning AND large language model AND manufacturing

**Table 3:** Inclusion and exclusion criteria for literature selection.

Criterion Type	Category	Description
Inclusion	Scope relevance	Studies explicitly involving FL and/or LLM within industrial–related scenarios.
	Technical depth	Works providing sufficient technical substance, including architectural descriptions, algorithmic design, methodological details, or empirical validation.
	Publication quality	Peer-reviewed journal articles and conference papers, as well as highly relevant preprints addressing emerging challenges in Fed-LLM.
Exclusion	Scope relevance	Studies focusing solely on FL or LLM without any industrial application context.
	Deployment feasibility	Studies that discuss FL or LLM only at an abstract or algorithmic level, without analyzing deployment feasibility under industrial constraints such as limited computation or communication resources, heterogeneity, privacy and security requirements.
	Research focus	Studies in which FL or LLM are mentioned merely as background concepts, auxiliary tools, or comparative baselines rather than as primary research objects.
	Publication type	Books, book chapters, technical reports, theses, dissertations, editorials, and other non-academic publications.

**Table 4:** Overview of C^2^ Overhead Fed-LLM Approaches.

Approach	Mechanism	Related Method
PEFT	By training and transferring only a small subset of parameters, computational and communication costs are reduced.	Adapter Tuning, Prefix-tuning, Prompt-tuning, and LoRA
Sparsification and Quantization	Sparsifying or quantizing parameters during computation or transmission, thus lowering computational demands and compressing communication bytes with minimal impact on convergence.	FLASC, RoLoRA, and FedPipe
Dynamic Adjustment and Adaptation	Optimizing communication timing, client involvement, and aggregation methods to dynamically scale local updates or minimize unnecessary communication.	SA-FedLoRA and FlexLoRA
Model Partitioning and Layered Training	Dividing models into layers or modules for collaborative training across clients or edge servers, synchronizing only a subset of layer parameters to reduce computational and communication overhead.	SplitLoRA, FedRA, and Fed-piLot

**Table 5:** Comparison of representative PEFT-based federated learning methods in terms of experimental setup and system design for industrial applicability.

Method	Type	FL-Setup	#Clients	Basic Model
FedAdapter [35]	Adapter	Cross-device	$10^{2}$–$10^{3}$	RoBERTa / LLaMA2-13B
FedTT+ [36]	Adapter	Cross-silo, Cross-device	10–$10^{3}$	DeBERTa-Base / LLaMA-2
FedPrefix [38]	Prefix	Cross-silo	64	ViT
PromptFL [42]	Prompt	Cross-device	64	CLIP
FedPepTAO [43]	Prompt, LoRA	Cross-device	100	LLaMA-7B
Fed-IT [46]	LoRA	Cross-device	100	LLaMA-7B
FedSA-LoRA [47]	Adaptive LoRA	Cross-device	10–100	LLaMA3-8B

**Table 6:** Comparison of privacy-preserving techniques for Fed-LLMs in industrial-scale scenarios. The number of check marks (✔) indicates the level or magnitude; more check marks represent higher values or stronger effects.

Techniques	Privacy	Accuracy Impact	C^2^ Cost	Scalability	Complexity
DP	✔	✔✔✔	✔	✔ ✔ ✔	O(n)
HE	✔✔✔	✔	✔✔✔✔	✔	$O(n^{2})$
SMPC	✔✔✔	✔	✔✔✔	✔ ✔	O(kn) where *k* = number of parties.

**Table 7:** Overview of heterogeneous Fed-LLM approaches. To address heterogeneity challenges, FL approaches typically require adaptive adjustments at the device, data, and model levels.

Challenge	Approach	Mechanism
Device Heterogeneity	PEFT	Clients train only lightweight adapters, LoRAs, and other fine-tunable modules, or dynamically adjust LoRA rank and adapter depth to adapt to heterogeneous computing power.
	Model Splitting & Partial Training	The global model is divided into layers or modules based on computational capabilities, with weaker devices training only the small modules assigned to them.
	Asynchronous Aggregation	Employs an asynchronous update mechanism, enabling high-performance clients to upload gradient updates more frequently.
Data Heterogeneity	Regularization	Incorporate global model constraints or parameter regularization terms into the local objective function.
	Adaptive Aggregation	Implement weighted strategies or adaptive averaging during the global aggregation phase.
	Client Clustered	Cluster clients based on data distribution similarity, share models within the same cluster, and optimize independently across different clusters.
	Meta-Learning	Employ meta-learning principles to learn global initialization parameters, enabling rapid adaptation to task distributions across clients.
	Multi-task Learning	Effectively models relationships between tasks to simultaneously learn multiple related tasks, enabling knowledge sharing and collaborative optimization.
Model Heterogeneity	Knowledge Transfer	Transfers knowledge to lightweight local student models via a global teacher model or client-side ensemble teachers.
	Subnetwork Extraction	Extract high-performance subnetworks through saliency scoring, pruning, or low-rank decomposition, updating only these critical parameters on the client.

**Table 8:** Comparison of representative Fed-LLM systems. These systems establish an engineering foundation for multi-party intelligent collaboration in Industry 4.0 scenarios.

Framework	Challenge					Multi-GPU	Benchmark
	C^2^ Overhead	Privacy and Security	Heterogeneity				
			Device	Data	Model		
FATE-LLM [100]	✔	✔	✔	✔	✔	✔	✘
FS-LLM [101]	✔	✔	✔	✔	✘	✔	✔
Shepherd [46]	✔	✘	✔	✔	✔	✘	✘
OpenFedLLM [102]	✔	✘	✔	✔	✘	✘	✔

## Data Availability

No new data were created or analyzed in this study.

## Abbreviations

**Abbreviation**	**Definition**
AI	Artificial Intelligence
IIoT	Industrial Internet of Things
CPS	Cyber-Physical System
LLMs	Large Language Models
SCM	Supply Chain Management
PdM	Predictive Maintenance
PLM	Product Lifecycle Management
FL	Federated Learning
Fed-LLM	Federated Large Language Model
PRISMA	Preferred Reporting Items for Systematic Reviews and Meta-Analyses
IoT	Internet of Things
NLP	Natural Language Processing
C^2^	Computation and Communication
PLCs	Programmable Logic Controllers
Non-IID	Non-Independent and Identically Distributed
PEFT	Parameter-Efficient Fine-Tuning
LoRA	Low-Rank Adaptation
KD	Knowledge Distillation
DP	Differential Privacy
HE	Homomorphic Encryption
SMPC	Secure Multi-Party Computation
AGV	Automated Guided Vehicle
RAG	Retrieval-Augmented Generation
